# Supplementary material for: Risk Factors for Lung Function Decline in Pediatric Asthma under Treatment: A Retrospective, Multicenter, Observational Study
Source: Children (Basel). 2022 Oct 4;9(10):1516. doi: 10.3390/children9101516 (PMC9600699; doi:10.3390/children9101516)
Supplement: Supplementary file 1 [file children-09-01516-s001.zip › children-1881687-supplementary.pdf]

Table S1. Numbers of visits/spirometry at each visit.

	Normal	Upward	Downward	Low
6-9 y.o	7 (1-43)	2.5 (1 – 29)	13 (1 – 43)	3.5 (1 – 23)
10-12 y.o	12 (1 – 52)	10 (1 – 35)	14 (1 – 33)	10 (3 – 21)
13-15 y.o	3 (1 – 38)	4 (1 – 25)	4 (1 – 33)	3 (1 – 22)

Median (range)

Table S2. Characteristics of the subjects who were included and excluded for analysis.

	Analyzed n=275	Excluded n=354	P value
Sex (boys), n (%)	169 (62)	227 (64)	0.492
Gestational age (weeks), median (range)	39 (38-40)	39 (38-40)	0.685
Birth weight (g), median (range)	3002 (2767-3258)	3035 (2716-3346)	0.517
Comorbid allergic diseases			
Atopic dermatitis, n (%)	135 (49)	160 (45)	0.311
Perennial allergic rhinitis, n (%)	160 (58)	203 (57)	0.792
Seasonal allergic rhinitis, n (%)	77 (28)	124 (35)	0.062
Food allergy, n (%)	129 (47)	139 (39)	0.041
Other comorbidities, n (%)	26 (17)	27 (8)	0.468
Family history and environment			
Parental asthma, n (%)	51 (20)	76 (23)	0.389
Parental smoking, n (%)	45 (33)	85 (43)	0.063
Pet ownership, n (%)	51 (24)	63 (26)	0.649
Laboratory data[#]			
Eosinophils (/μL), median (range)	500 (300-758)	490 (290-790)	0.429
Total-IgE (IU/mL), median (range)	921 (415-1663)	807 (318-1661)	0.325
HDM-sIgE (kU_A/L), median (range)	97.9 (27.5-136)	76.7 (20.4-125)	0.138
JCP-sIgE (kU_A/L), median (range)	30.8 (4.1-100)	21.9 (4.2-84.7)	0.322
Asthma treatment before 6 years old			
ICS	151 (59)	196 (62)	0.531
LTRA	208 (81)	259 (83)	0.643
